# Death Rate

**Published:** 1859-08

**Authors:** 


### DEATH RATE

Is the term given to the proportion of persons dying in one year, in a given number. The lowest death rate ascertained with any certainty in the world, is in the isolated islands of Faroe, where fifteen persons die annually out of every thousand. In New York, one of the most, if not the most sickly city in the world, the last reported year of 1857, gave twenty-three thousand three hundred and thirty-three deaths. Thus, at the usually estimated population of that year, thirty-one persons died out of every thousand; more than double the mortality of the ignorant, thriftless and degraded Faroes. In the most notorious districts of England, the highest rate of mortality is thirty-six persons out of a thousand in one year. Whatever number of persons over fifteen die out of a thousand in a year, that is a mortality greater than ought to be. It therefore follows, that in New York city, which is more favorably situated for good drainage and cleanliness and ventilation than any other large city on the globe, two persons die where there ought to be only one; and where there would be but one, if paid and sworn officials did their duty, and private people lived wisely.

